# Cost-effectiveness of uterine balloon tamponade devices in managing atonic post-partum hemorrhage at public health facilities in India

**DOI:** 10.1371/journal.pone.0256271

**Published:** 2021-08-18

**Authors:** Beena Nitin Joshi, Siddesh Sitaram Shetty, Kusum Venkobrao Moray, Oshima Sachin, Himanshu Chaurasia

**Affiliations:** 1 Department of Operational Research, Indian Council of Medical Research - National Institute for Research in Reproductive Health, Mumbai, Maharashtra, India; 2 Regional Resource Hub for Health Technology Assessment, Indian Council of Medical Research - National Institute for Research in Reproductive Health, Mumbai, Maharashtra, India; 3 Department of Health Research, Ministry of Health and Family Welfare, New Delhi, India; Icahn School of Medicine at Mount Sinai, UNITED STATES

## Abstract

**Objective:**

Post-partum hemorrhage (PPH) is the leading direct cause of maternal mortality in India. Uterine balloon tamponade (UBT) is recommended for atonic PPH cases not responding to uterotonics. This study assessed cost-effectiveness of three UBT devices used in Indian public health settings.

**Methods:**

A decision tree model was built to assess cost-effectiveness of Bakri-UBT and low-cost ESM-UBT alternatives as compared to the recommended standard of care i.e. condom-UBT intervention. A hypothetical annual cohort of women eligible for UBT intervention after experiencing atonic PPH in Indian public health facilities were evaluated for associated costs and outcomes over life-time horizon using a disaggregated societal perspective. Costs by undertaking primary costing and clinical parameters from published literature were used. Incremental cost per Disability Adjusted Life Years (DALY) averted, number of surgeries and maternal deaths with the interventions were estimated. An India specific willingness to pay threshold of INR 24,211 (USD 375) was used to evaluate cost-effectiveness. Detailed sensitivity analysis and expected value of information analysis was undertaken.

**Results:**

ESM-UBT at base-case Incremental Cost-Effectiveness Ratio (ICER) of INR -2,412 (USD 37) per DALY averted is a cost-saving intervention i.e. is less expensive and more effective as compared to condom-UBT. Probabilistic sensitivity analysis however shows an error probability of 0.36, indicating a degree of uncertainty around model results. Bakri-UBT at an ICER value of INR -126,219 (USD -1,957) per DALY averted incurs higher incremental societal costs and is less effective as compared to condom-UBT. Hence, Bakri-UBT is not cost-effective.

**Conclusion:**

For atonic PPH management in India, condom-UBT offers better value as compared to Bakri-UBT. Given the limited clinical effectiveness evidence and uncertainty in sensitivity analysis, cost-saving result for ESM-UBT must be considered with caution. Future research may focus on generating high quality comparative clinical evidence for UBT devices to facilitate policy decision making.

## Introduction

Investment in maternal health improves health outcomes and substantially benefits overall socio-economic development of the society [1–3]. Though maternal health financing towards life-saving interventions has steadily improved over time, key factors like sustainable and efficient funding, pertinent health policies and a reliable supply of life saving commodities ensuring coverage of health services has still fallen short [4]. Efficient use of available resources by implementing cost-effective interventions can prevent maternal morbidity and mortality. Post-partum hemorrhage (PPH) is defined as maternal blood loss of 500 ml or more within 24 hours after delivery and is responsible for two-thirds of global maternal deaths occurring due to hemorrhage [5, 6]. Obstetric hemorrhage is the leading direct cause of maternal deaths worldwide. Skilled healthcare providers, timely referrals, availability of emergency obstetric services and a reliable supply of life-saving commodities can avoid these deaths occurring due to PPH [3, 7].

PPH affects nearly 3-6% of all women giving birth in India [8, 9]. Failure of uterus to contract after delivery i.e. uterine atony is the commonest cause of PPH [10]. India accounts for one-fifth of all maternal deaths globally with PPH as its leading cause [11, 12]. Management in India begins by preventing PPH using active management with uterotonics, cause-specific PPH treatment, resuscitation for shock and patient referral to higher facilities for further treatment. For atonic PPH, uterotonics remain the mainstay treatment in accordance with the World Health Organization (WHO) guidelines [6]. The WHO recommends using Uterine Balloon Tamponade (UBT) intervention for atonic PPH cases that do not respond to first-line treatment after vaginal delivery provided recourse to surgical interventions, access to blood products, skilled/trained health personnel, resources and treatment protocols are in place [13]. The latest WHO guidance has identified that existing evidence for UBT intervention is disparate however has recognized that it is probably feasible and acceptable to women and healthcare providers in contexts where treatment protocols are available and implemented. A recently concluded systematic review of ninety-one studies has also suggested that UBT use is associated with significant reduction in PPH related invasive procedures [14]. The review reported a pooled UBT success rate of 85.9% in controlling PPH. Although UBT intervention was highly successful in controlling severe PPH bleeding, efficacy and effectiveness evidence between experimental and observational studies was found conflicting with quality of evidence identified as a limitation. Two RCT studies included in the systematic review saw no benefit of introducing UBT in refractory PPH management [15, 16]. The respective studies however reported significant associated limitations and biases impacting their results. A recent Cochrane review has concluded that evidence from existing RCT studies is insufficient to determine relative effectiveness of mechanical and surgical interventions in treating primary PPH [17]. The latest WHO guidelines acknowledges that UBT recommendation may not be operationalized in a standard and consistent manner across different settings. For the diverse Indian health system with initiatives such as the ‘Dakshata’ and ‘LaQshya Guidelines’ that focus on standardization of institutional deliveries across India by adhering to clinical protocols for management and equipping providers for comprehensive maternal health care, UBT intervention for atonic PPH management as a relatively simple technique can be a life-saving intervention [18]. UBT can potentially avoid need for surgery and even act as a temporizing measure while awaiting transfer to the higher center.

Multiple UBT devices specifically designed or assembled for use in PPH management are presently available. Indian guidelines recommend using condom-UBT device for management of refractory atonic PPH [11]. Condom-UBT is an improvised device assembled at service delivery point using readily available components like a male condom, Foley catheter, string/suture and intravenous (IV) infusion set. The cost of assembly components of condom-UBT device or its modifications available in public health facilities of India is very low, estimated at Indian National Rupee (INR) 128 (USD 2). Bakri balloon and Every Second Matters (ESM) UBT, two United States Food and Drug Administration (US-FDA) approved devices specifically designed for PPH management are also being used across a few public health settings in India [19–21]. Bakri-UBT comes in a sterile pack, is ready to use and has a drainage outlet to measure ongoing blood loss. At a market price of INR 9,554 (USD 148), Bakri-UBT is commercially available in India but is quite expensive [22]. ESM-UBT, a low-cost alternative with documented mechanical properties and assembly components available in a sterile pack with an instruction manual is relatively inexpensive at INR 397 (less than USD 5), but is commercially not available in India at present [14, 23, 24].

India provides free treatment for post-natal complications of pregnancy to women delivering at public health facilities under the *Janani Shishu Suraksha Karyakaram* (JSSK) scheme [25]. Given the ever growing need for resource prioritization, decision making that is evidence informed is becoming increasingly important. In this regard to reduce health opportunity costs, India is generating cost-effectiveness evidence as a tool to prioritize public health interventions. Choosing a specific UBT device for PPH complication management has associated health and economic consequences. Evidence on economic perspective of UBT intervention is limited globally. A recent systematic review analyzing cost-effectiveness of UBT intervention found only two studies that had evaluated its cost-effectiveness [26]. A Kenyan modelling study found ESM-UBT to be highly cost-effective for severe PPH management [27]. Another study from year 2006 evaluating economic efficiency of various interventions in reducing PPH across four developing countries including India observed UBT to be the most-cost effective alternative among curative options for PPH management. The assessment however identified UBT intervention to not be adequately researched and that cost-effectiveness findings were based on clinical effect estimates from two small case series studies [28]. The systematic review concluded that evidence on cost-effectiveness of UBT intervention is limited and not generalizable to different contextual settings.

The growing clinical evidence for individual UBT devices has not yet been used to evaluate and compare multiple UBT device alternatives using a cost-effectiveness approach to inform decision-making. This present study aimed to undertake an economic evaluation for UBT intervention by determining the most cost-effective UBT device for atonic PPH management in the Indian context. This study will help policy makers choose a UBT device that offers the highest value. Objective of this study was to assess cost-effectiveness of condom-UBT, ESM-UBT and Bakri-UBT devices in managing atonic PPH across public health facilities of India.

## Methods

### Model overview

A decision tree type of decision analytic model was built to estimate the expected costs and consequences of using ESM-UBT or Bakri-UBT device as compared to standard care i.e. condom-UBT in atonic PPH management. Globally, Bakri-UBT is one of the most commonly used UBT devices. ESM-UBT is being assessed for introduction in the Indian public health system as a low-cost alternative. As UBT intervention for atonic PPH management is a discrete event occurring over a short duration without any recurring events or health state transitions, a decision tree model was considered appropriate. As recommended in India, disaggregated societal perspective was used to analyze costs and effects of the intervention [29]. This comprised of public health system costs in delivering UBT intervention and out-of-pocket expenditure (OOPE) incurred by households at the time of childbirth. Effects were measured in terms of Disability Adjusted Life Years (DALY) averted and Incremental Net Monetary Benefit (NMB) of the intervention [30]. Given the high burden of PPH in India, nature of the condition, availability of India inclusive disability weights for maternal haemorrhage from the Global Burden of Disease report, DALYs were chosen as the primary outcome measure [31]. Net monetary benefit framework has an advantage in interpreting negative Incremental Cost-Effectiveness Ratio (ICER) values and hence was used in the analysis. The modeled base-case population included a cohort of Indian women with median age 21 years at first child birth accessing public health facilities for atonic PPH over one-year study implementation duration (2017-18) [32]. Specifically, UBT intervention lasts for a short duration with highest reported mean UBT device retention time of 27.5 hours across reviewed studies [33]. Though PPH event lasts for a short time, consequences or outcomes as a result of the condition or its clinical management may likely result in long-term consequences. This includes disabilities due to hysterectomy, associated infertility or maternal death after PPH event. These disabilities may get further exacerbated for a healthcare setting like India wherein health infrastructure or expertise for conservative surgical procedures may not always be readily available across settings. A life-time horizon was thus considered appropriate to account for consequences of using UBT intervention. A standard three percent discount rate was used to account for all primary costs and model outcomes [34, 35]. As costs incurred for UBT intervention are expected to be non-recurring and a one-time event, estimated costs in the model were kept undiscounted. To account for health outcomes occurring over life-time horizon as a result of the intervention, model outcomes were discounted.

### Model structure

**Fig 1:**
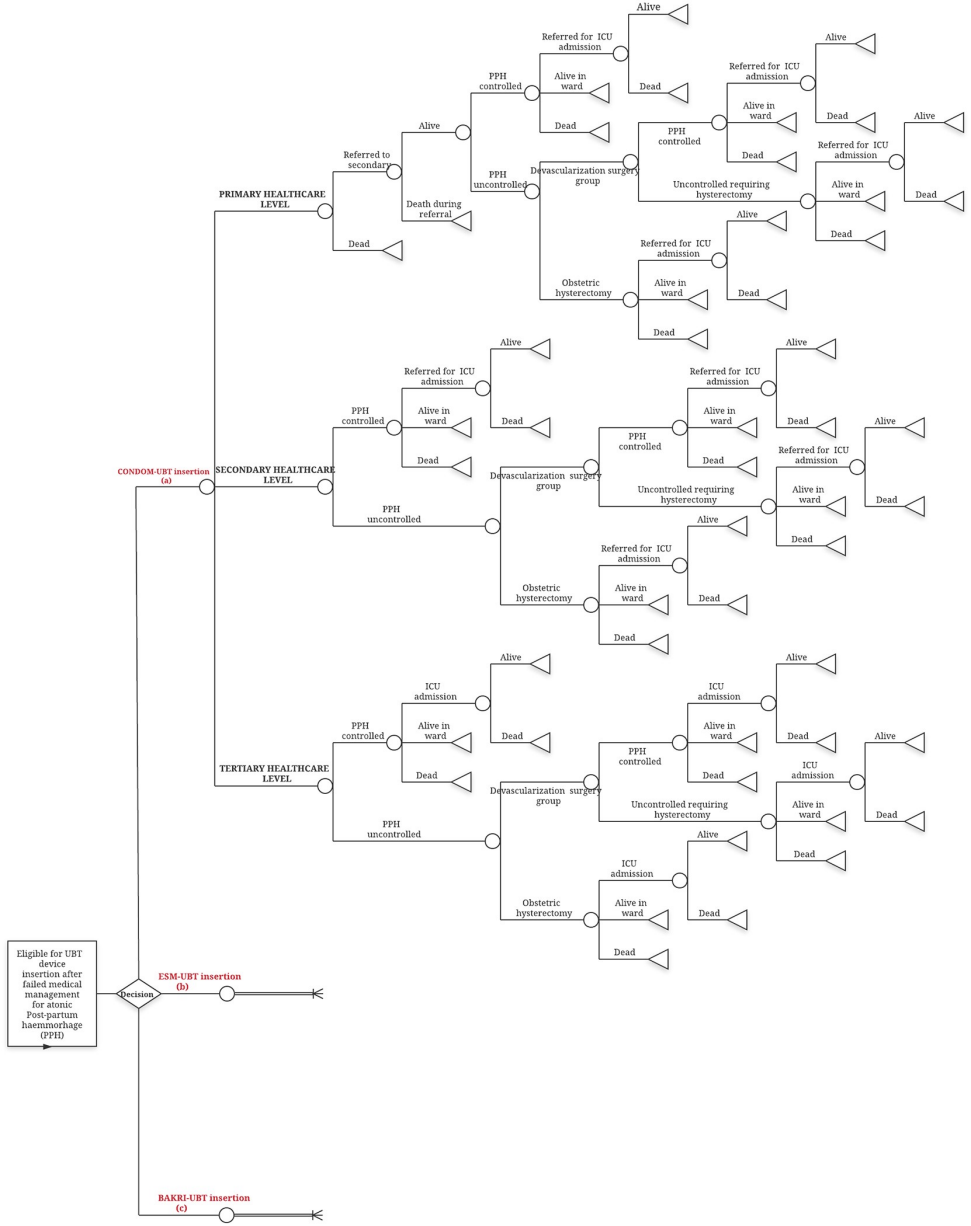
Decision tree model for atonic PPH management with UBT intervention in Indian public health settings. A decision is made regarding the choice of UBT device used for eligible women at public healthcare levels in India experiencing atonic PPH. Pathway for scenario with ESM-UBT or Bakri-UBT is the same as depicted for condom-UBT. Circles represent chance nodes. Triangles represent terminal nodes indicating end of the decision tree pathway.

The model was built using Microsoft Excel 2016 with Microsoft Visual Basic for Application 7.1. The decision tree as described in Fig 1, begins with a cohort of women delivering in public health facilities of India experiencing atonic PPH that does not respond to uterotonics thereby becoming eligible for UBT insertion. A decision at this point is made regarding choice of UBT device used for management. The pathway followed from this decision node depends on services available at the health facility level accessed by women and clinical course of the condition.

For the decision tree as described in Fig 1, public health facilities in India were classified into three levels based on services provided for PPH management as per Indian guidelines [11, 36, 37]. Primary level care for PPH comprises of Primary Health Centre (PHC) facility that is equipped with medical officer and skilled birth attendants to initially stabilize, insert UBT device and refer women to a higher facility. Secondary level care consists of Community Health Centers (CHC) and Sub-District Hospitals (SDH) additionally equipped with Obstetrics-Gynecology (OBGYN) specialist, Operation Theater (OT) and blood transfusion facilities. Tertiary level care for PPH includes additional advanced intervention and Intensive Care Unit (ICU) facility available at District Hospitals (DH) and medical colleges. Patients from secondary level care requiring ICU facilities are expected to be referred.

UBT device insertion is considered after uterotonics like intramuscular or IV oxytocin, IV methyl-ergometrine, sublingual misoprostol fails to control atonic PPH bleeding. Resuscitation measures for shock prevention or management are continued. Cases remaining uncontrolled after UBT insertion would undergo surgical intervention like B-Lynch compression suturing, stepwise devascularization surgery or directly a life-saving obstetric hysterectomy depending on woman’s clinical condition and availability of resources at facility. A patient uncontrolled after conservative surgical procedures may also eventually require hysterectomy procedure to control bleeding. Surgical facilities are expected to be available at both secondary and tertiary levels. PPH cases with severe blood loss, post-surgery or any other complications may need ICU facility for management available at tertiary level. A woman may die at any point during the course either due to atonic PPH complication or other maternal mortality causes.

Certain assumptions were made in decision tree conceptualization. First, it was assumed that all eligible women would receive one common UBT device across all facilities in the Indian public health system. In ESM-UBT scenario for instance, it was assumed that all eligible women would exclusively receive ESM-UBT device across the public health system and so on for the other two devices. Secondly, it is assumed that all women accessing primary level facilities would be referred after UBT insertion and initial stabilization. An effective system for immediate referral of patients from primary level exclusively to secondary level and from secondary level to tertiary care for ICU service is assumed to be in place. Thirdly, conservative surgeries like B-lynch suturing and stepwise devascularization are grouped together as a single surgical procedure in the model.

### Model inputs

#### 1. UBT device effectiveness

Published literature was collated to assess clinical effectiveness of individual UBT devices evaluated in this study. Effectiveness was defined as control of atonic PPH bleeding after UBT device insertion measured directly or as surgical interventions/maternal deaths averted to control bleeding after UBT insertion. As ESM-UBT device was the intervention being assessed for introduction, a systematic review was undertaken after registering with PROSPERO 2019 database (Registration number CRD42019122802) [38]. Only three published ESM-UBT case-series studies were identified to be eligible for the systematic review. For condom-UBT and Bakri-UBT, a targeted review was undertaken by searching three electronic databases for published literature between January 2010 to December 2019. Studies evaluating effectiveness of condom-UBT and Bakri-UBT in atonic PPH management, available in English language were included. For targeted review, a total of 30 articles comprising twelve condom-UBT and nineteen Bakri-UBT studies, one of which was a comparative randomized control trial (RCT) study between the two devices were eventually included. Overall eligible reviewed studies for the three UBT devices comprised of one systematic review, three RCTs and twenty-six prospective or retrospective studies largely indicating low quality of evidence across hierarchical structure. Primary outcome considered by the limited available ESM-UBT studies was survival rates in contrast to the other two UBT devices wherein majority studies reported control in PPH bleeding. The mean summary measure of effectiveness for each individual UBT type was computed from effectiveness rates reported by the studies. All condom, ESM-UBT and nearly two-fifth Bakri-UBT studies reviewed for this analysis reported findings from developing country settings. S1 Table reports the identified eligible studies reviewed to ascertain clinical effectiveness of the three UBT devices.

Clinical and epidemiological parameters used in the model were obtained from published literature and facility level Health Management Information System (HMIS). Data on public healthcare facility accessed for childbirth was obtained from National Family Health Survey (NFHS-4), India [32]. As specific data for UBT use in PPH across healthcare facilities was unavailable, probability estimates for incidence of PPH (3.6% of deliveries), atonic PPH incidence (80% of PPH cases), control of cases with medical treatment (90% cases), probability of specific surgical intervention, surgery success rate and morbidity data associated with PPH as reported in Table 1 were obtained from literature relevant to the Indian context [8, 9, 39–41]. Though the comparative RCT (Condom-UBT versus Bakri-UBT) had some parameters that could have been used in model, the study sample size was very small and no statistically significant differences were observed between the two UBT devices. So, studies with large samples specific to healthcare services in the Indian context were used to compute model parameters wherever available. Probability of PPH and all-cause mortality was calculated using data from the Census of India, Sample Registration Survey [42]. A list of all parameters including UBT effectiveness rates, epidemiological and clinical parameters used in the model are reported in Table 1.

**Table 1:** Input parameters used in the model with upper and lower limits.

Input parameter	Base-case value	Lower limit	Upper limit	Source (Reference)
**Demographic and epidemiological parameters**				
Median age of onset	21	16.8	25.2	[32]
Deliveries in Indian public health facility (Includes home deliveries)	20,785,669	16,628,535	24,942,803	[32]
Probability of all cause maternal mortality in India	0.00308	0.00246	0.00369	Calculated from [42]
Probability of maternal mortality due to PPH in India	0.00108	0.00086	0.00130	Calculated from [42]
Proportion of deliveries at primary healthcare level in India	0.18610	0.14888	0.22332	[32]
Proportion of deliveries at secondary healthcare level in India	0.32930	0.26344	0.39516	[32]
Proportion of deliveries at tertiary healthcare level in India	0.48460	0.38768	0.58152	[32]
Incidence of post-partum haemorrhage in India/	0.03606	0.02885	0.04327	Calculated from [8, 43]
Annual cohort of women eligible for UBT device insertion after experiencing atonic PPH	59,962	47,970	71,955	Calculated form [8, 39, 40, 43]
**Clinical parameters**				
Proportion of atonic PPH cases controlled after condom-UBT insertion	0.923	0.738	0.983	Estimated from targeted literature review
Proportion of atonic PPH cases controlled after Bakri-UBT insertion	0.843	0.674	0.983	Estimated from targeted literature review
Proportion of atonic PPH cases controlled after ESM-UBT insertion	0.953	0.762	0.983	Estimated from systematic literature review
Proportion of uncontrolled atonic PPH cases undergoing obstetric hysterectomy immediately after UBT insertion	0.14634	0.11707	0.17561	Calculated from [40]
(Based on patient’s clinical condition, severity of PPH bleeding and health system resources)				
Proportion of uncontrolled atonic PPH cases undergoing obstetric hysterectomy after devascularization group of surgery	0.21951	0.17561	0.26341	Calculated from [40]
(Undertaken as a life-saving intervention if bleeding remains uncontrolled despite using conservative surgical procedures)				
Probability of ICU admission in atonic PPH cases successfully controlled with UBT insertion	0.025	0.020	0.030	[41]
Probability of ICU admission in atonic PPH cases uncontrolled after UBT insertion	0.769	0.61520	0.92280	[41]
**Cost parameters (INR)**				
Condom-UBT insertion cost at primary healthcare level (Includes device cost of INR 128)	161	97	226	[44]
Condom-UBT insertion cost at secondary healthcare level (Includes device cost of INR 128)	341	264	419	
Condom-UBT insertion cost at tertiary healthcare level (Includes device cost of INR 128)	419	342	510	
ESM-UBT insertion cost at primary healthcare level (Includes device cost of INR 397)	432	232	639	
ESM-UBT insertion cost at secondary healthcare level (Includes device cost of INR 397)	567	381	748	
ESM-UBT insertion cost at tertiary healthcare level (Includes device cost of INR 397)	671	548	806	
Bakri-UBT insertion cost at primary healthcare level (Includes device cost of INR 9554)	9,585	4,792	14,125	
Bakri-UBT insertion cost at secondary healthcare level (Includes device cost of INR 9554)	9,746	5,676	13,855	
Bakri-UBT insertion cost at tertiary healthcare level (Includes device cost of INR 9554)	9,874	7,288	12,371	
Devascularization surgery cost at secondary level care after ESM-UBT insertion	3,671	2,393	5,095	
Obstetric hysterectomy cost at secondary level care after ESM-UBT insertion	7,734	5114	10,494	
Devascularization surgery cost at tertiary level care after ESM-UBT insertion	3,335	2,618	4,108	
Obstetric hysterectomy cost at tertiary level care after ESM-UBT insertion	5,579	4,386	6,908	
Devascularization surgery cost at secondary level care after Condom-UBT insertion	4,864	3,186	6,733	
Obstetric hysterectomy cost at secondary level care after Condom-UBT insertion	7,788	5,025	10,849	
Devascularization surgery cost at tertiary level care after Condom-UBT insertion	3,418	2,703	4,154	
Obstetric hysterectomy cost at tertiary level care after Condom-UBT insertion	5,470	4,276	6,734	
Devascularization surgery cost at secondary level care after Bakri-UBT insertion	4,954	3,302	6,701	
Obstetric hysterectomy cost at secondary level care after Bakri-UBT insertion	7,721	5,166	10,668	
Devascularization surgery cost at tertiary level care after Bakri-UBT insertion	3,418	2,683	4,173	
Obstetric hysterectomy cost at tertiary level care after Bakri-UBT insertion	5,470	4,334	6,688	
ICU admission cost at tertiary level care for atonic PPH management	4,896	3,244	6,746	
Inpatient admission cost at secondary level care for atonic PPH management	1,774	1,083	2,548	
Inpatient admission cost at tertiary level care for atonic PPH management	1,806	1,335	2,315	
Health system cost for referral of a patient	1,001	801	1,201	[45]
Healthcare provider training cost per patient	375	300	450	Estimated using unpublished resources, expert opinion and assumptions
Out-of-pocket expenditure for childbirth	2,755	2,204	3,306	[43]
**Other parameters**				
Discount rate	0.030	0.000	0.050	[29]
Disability weight for maternal haemorrhage (Less than 1-liter blood loss)	0.114	0.078	0.159	[31]
Disability weight for maternal haemorrhage (Greater than 1-liter blood loss)	0.324	0.220	0.442	[31]
Disability weight for infertility due to PPH (Proxy weight of infertility due to puerperal sepsis)	0.005	0.002	0.011	[31]

PPH-Postpartum Haemorrhage, UBT – Uterine Balloon Tamponade, ESM- Every Second Matters, ICU – Intensive Care Unit, INR – Indian National Rupee

#### 2. Costs

A primary bottom-up economic micro-costing study with a convenience sample of one PHC representing primary care, one SDH representing secondary care and one DH and a government medical college hospital representing tertiary level care in the state of Maharashtra was undertaken to asses cost of managing atonic PPH in Indian public health settings for the year 2017-18 [44]. Costs were calculated specifically for condom-UBT intervention i.e. standard of care with similar estimation for ESM-UBT and Bakri-UBT using respective clinical effectiveness and UBT device price. Cost estimates for individual UBT type with subsequent interventions from primary costing was then used in the decision tree. Cost components in the decision tree included unit cost of UBT insertion, devascularization surgery, hysterectomy, inpatient department (IPD) admission and ICU admission for a PPH event at respective healthcare levels. Unit cost of UBT insertion included UBT device price in addition to the economic cost of resource utilization in the form of human resources, area, equipment, drugs and consumables and overhead utilities. UBT insertion costs were calculated respectively for both labor room and operation theatre to get a weighted aggregate facility level unit cost. This was followed by aggregation of DH and medical college costs respectively to get unit cost for primary, secondary and tertiary healthcare levels. Inpatient admission cost for a unit PPH event was estimated by calculating per-day facility cost for an obstetric patient admitted at the health facility for an average of 3.4 days followed by apportioning to 2.98 days as reported for atonic PPH stay [46]. ICU cost for PPH event admission was similarly estimated by calculating per-day ICU admission cost of an obstetric patient admitted in the ICU for an average of 3.47 days followed by apportioning to the reported 1.5 days for PPH admission [41, 47]. As PPH related referral data was unavailable during analysis, referral cost was obtained from a published Indian study reporting public health system cost of referrals related to institutional deliveries after making inflation adjustment to the year 2017-18 [45, 48]. All respective unit costs were applied to the cohort of atonic PPH cases in India eligible for UBT intervention run through the decision tree pathway. Costs in this study are reported in Indian National Rupee (INR) and United States Dollars (USD) currency. A conversion rate of 1 USD = 64.5 INR for the year 2017-18 was used in cost analysis [49].

Certain costing assumptions were made for the decision model. Cost of blood transfusion and fluid resuscitation were not considered separately and are incorporated in unit costs. A one-time annual training of healthcare providers for UBT intervention irrespective of the type of device was assumed and costs were estimated.

Disaggregated societal costs for the decision tree was obtained by combining health system costs, an annual training and OOPE incurred by the households. OOPE was obtained from the 75^th^ round of National Sample Survey 2017-18 [43]. NSS survey encompasses a very large number of rural and urban Indian households and considers both medical and non-medical expenditures such as food, transport, escort expenditures, lodging, etc. incurred by households on account of childbirth. Table 1 presents the input and assumption parameters used in costing analysis.

#### 3. Valuation of consequences

The health consequences of using any of the three UBT devices after atonic PPH event in this study was estimated using DALY outcome measure. UBT intervention contributes to subsequent reduction in need for surgeries, thus reducing maternal morbidity and mortality [14]. Even with UBT intervention, surgeries like devascularization, obstetric hysterectomy and complications related to PPH may still occur depending on patient’s clinical response and type of UBT device used. To ascertain such associated outcomes with each UBT type, number of surgeries and maternal deaths with the three UBT devices were also estimated using event probabilities. DALY as an outcome measure summarizes disease burden by combining both time lost through premature death and time lived in a state of less than full health labelled as disability. DALYs were estimated by calculating Years of Life Lost (YLL) and Years Lived with Disability (YLD) subsequent to UBT intervention for PPH condition using the simplified DALY estimation method [50].


DALY=YLL+YLD

YLL=Numberofdeaths*lifeexpectancyatageofdeath

$$\text{YLD}=\text{Number}\;\text{of}\;\text{cases}*\text{average}\;\text{duration}\;\text{until}\;\text{remission}\;\text{or}\;\text{death}\left(\text{years}\right)*\text{disability}\;\text{weight}$$

For DALY estimation over life-time horizon, YLL or YLD were calculated at each respective terminal node of the decision pathway. At terminal nodes for PPH event, patients are expected to remain alive with associated disability due to PPH event or its consequences or may die either due to bleeding or other causes of maternal mortality. YLL for premature death due to PPH event was calculated using age-specific life-expectancy of the Indian female population. For the considered hypothetical cohort of Indian women aged 21 years dying due to PPH after childbirth, remaining age-specific life expectancy of 53.79 years from abridged life-tables determined YLLs for deaths in the decision tree [51]. For YLD of those remaining alive, patients having bleeding controlled with UBT intervention alone were assigned disability weight of mild maternal hemorrhage (less than 1-liter blood loss) whereas remaining cases undergoing conservative surgery, obstetric hysterectomy or ICU admission for PPH control were assigned the disability weight of severe maternal hemorrhage (greater than 1-liter blood loss) obtained from Global Burden of Disease study [52]. Additionally, those undergoing hysterectomy for PPH control were assigned the disability weight of secondary infertility for their remaining reproductive life-span. As disability weight for infertility due to obstetric hysterectomy after PPH was unavailable, we assumed infertility due to puerperal sepsis from GBD study to be an appropriate estimate. Time duration of disability due to hemorrhage was assumed to last for a postpartum period of six weeks (0.11 years) whereas in cases of hysterectomy, additional disability weights of secondary infertility were assigned for the remaining reproductive life-span up to 46^th^ year for the study cohort [53, 54]. Number of cases at each terminal node was obtained as the product of probability of events at each preceding node leading to the terminal node. Number of cases at each terminal node was then multiplied with remaining expected life-expectancy for YLLs or outcome specific disability weights for specific defined duration of disability to determine YLDs at terminal nodes. Overall DALYs associated with UBT intervention was thus estimated by combining YLL and YLDs. As an example of DALY calculation, for the estimated number of women having atonic PPH bleeding controlled with hysterectomy after failed medical, UBT and conservative surgical interventions, disability weight of severe hemorrhage for duration of 0.11 years and disability weight of hysterectomy associated secondary infertility for a reproductive time-span of 25 years determined YLD for this terminal node. Similarly, for women remaining alive after control of PPH bleeding with UBT intervention alone, disability weight of mild hemorrhage was assigned for a duration of 0.11 years to determine YLD for this node. DALY calculation in this study did not consider age weighting. A discount rate of three percent to account for future DALY outcomes over life-time horizon as recommended by the Indian reference case was considered in base-case analysis [29]. Table 1 presents the input parameters for PPH consequences used in the model.

### Cost-effectiveness

Incremental cost-effectiveness ratio (ICER) i.e. incremental costs per DALY averted for ESM-UBT versus condom-UBT and Bakri-UBT versus condom-UBT comparisons was calculated. A simplified net monetary benefit framework (Incremental NMB = Incremental benefit*Willingness-to-pay (WTP) threshold – Incremental costs) was estimated for the interventions against comparator i.e. standard care. To determine cost-effectiveness, an India specific WTP was obtained from a study that empirically calculated country specific cost per DALY averted threshold for 97 different low-income and middle-income countries [55]. The study calculated threshold value using India specific health expenditure, survival and morbidity burden. An inflation adjusted Indian value of INR 24,211 (USD 375) per DALY averted for year 2017 was chosen as the threshold for this study [56, 57]. As sensitivity analysis evaluates interpretation of higher WTP such as that recommended by the WHO, the chosen lower threshold was considered appropriate for base-case analysis [58].

### Sensitivity analysis

Sensitivity analysis was used to address uncertainties in the model. Probabilistic sensitivity analysis (PSA) was used to evaluate joint parameter uncertainty effect. Evidence base used for clinical effectiveness of UBT devices was of limited strength with only three studies evaluating ESM-UBT intervention. For this reason, distribution of clinical effectiveness parameter in sensitivity analysis for each UBT device was varied by 20 percent limit at lower end and a ceiling 98 percent (highest reported effectiveness for UBT from literature review) for all comparators. Primary cost parameters used in the model were varied along the 95 percent Confidence Interval (CI) limits derived by running 1,000 Monte Carlo simulations for each unit cost estimate obtained. Parameters of each unit cost were assigned distributions and then varied at both ends by assuming a variation of 50 percent for UBT device price to account for expectation of cost variability, a 50 percent upper and 100 percent lower limit respectively for drugs and consumables given the government procurement at a negotiated price and a 25 percent variation for remaining parameters like human resource salaries, area price, equipment (medical/non-medical), utilities and utilization of resources. For model, remaining cost parameters like referral cost, training and OOPE were varied by a 20 percent assumption at both ends. Disability weights were varied along CI limits provided by the GBD study. Discount rates were varied between 0 to 5 percent as recommended for India [29]. Remaining model parameters were varied by 20 percent on either side of the base-case value. A Beta distribution was assigned to all probabilities, proportions and disability weight parameters whereas a Gamma distribution was used for costs and resources. To determine influence of each individual model parameter on cost-effectiveness results, One-Way Sensitivity Analysis (OWSA) was undertaken using the fore mentioned distributions. Tornado diagram was used to graphically represent the most influential parameters in the model. Monte Carlo method was used to compute 10,000 PSA simulation results for the model. An ICER (DALY) plane and a Cost-Effectiveness Acceptability Curve (CEAC) at different WTP values was used to represent uncertainty using probabilistic sensitivity analysis results. Table 1 presents the base-case input values used in sensitivity analysis with their upper and lower limits.

The model was validated using the AdViSHE tool for health economic models and the study was reported using the ISPOR-CHEERS checklist [59, 60]. S1 Appendix provides the checklist tools. The study was approved by the Ethics Committee for Clinical Studies at National Institute for Research in Reproductive Health, Mumbai under approval number D/ICEC/Sci-29/31/2018. State health department administrative approvals and consent from respective health facilities were obtained to undertake primary costing study.

## Results

### Costs

An estimated 59,962 women accessing public health facilities across India in the year 2017-18 were eligible for UBT device insertion after failure of medical management in controlling atonic PPH bleeding. Management of these eligible cases using condom-UBT, ESM-UBT or Bakri-UBT device has an associated disaggregated societal cost of INR 380,023,259 (USD 5.89 million), INR 375,629,967 (USD 5.82 million) or INR 993,068,492 (USD 15.39 million) respectively. Of this, the public health system incurs a cost of INR 192,277,700 (USD 2.98 million), INR 187,880,533 (USD 2.91 million) or INR 805,236,204 (USD 12.4 million) for using the three respective devices. The estimated societal unit cost per case is INR 6,338 (USD 98) for condom-UBT, INR 6,264 (USD 97) for ESM-UBT and INR 16,561 (USD 257) for treatment using Bakri-UBT device along with other surgical interventions for remaining uncontrolled cases after UBT device insertion. Using ESM-UBT instead of the currently recommended condom-UBT is associated with an incremental societal cost-saving of INR 73 (USD 1.1) per atonic PPH case. Similarly, using Bakri-UBT as compared to condom-UBT device results in an incremental societal cost-spending of INR 10,224 (USD 158.5) per eligible atonic PPH case. Costs of managing atonic PPH with the three UBT devices in Indian public health system along with upper and lower limit range is presented in Table 2.

**Table 2:** Costs and outcomes with UBT devices for atonic PPH management in Indian public health system.

Characteristics	Condom-UBT (Standard of care)	ESM-UBT	Bakri-UBT
Total societal cost in INR (Range) [USD]	380,023,259 (278,456,845 - 525,081,934)	375,629,967 (275,009,630- 547,823,882)	993,068,492 (587,281,814 - 1,443,326,391)
	[5,891,833 (4,317,160 – 8,140,960)]	[5,823,720 (4,263,715 – 8,493,394)]	[15,396,411(9,105,144 - 22,377,153)]
Total health system cost in INR (Range) [USD]	192,277,700 (158,310,666 - 254,701,345)	187,880,533 (154,864,947 - 277,426,632)	805,236,204 (467,116,489 - 1,172,706,867)
	[2,981,050 (2,454,429 - 3,948,858)]	[2,912,876 (2,401,006 - 4,301,188)]	[12,484,282 (7,242,116 - 18,181,502)]
Per patient societal cost in INR (Range) [USD]	6,338 (4,644 - 8,757)	6,264 (4,586 - 9,136)	16,561(9,794 - 24,070)
	[98 (72 -136)]	[97 (71 - 142)]	[257 (152- 373)]
Per patient health system cost in INR (Range) [USD]	3,207 (2,640 - 4,248)	3,133 (2,583 - 4,627)	13,429 (7,790 - 19,557)
	[50 (41- 66)]	[49 (40 - 72)]	[208 (121 - 303)]
Discounted DALYs per patient (Range)	0.2156	0.1852	0.2966
	(0.1497 - 0.50)	(0.1497 - 0.48)	(0.1497 - 0.58)
Total maternal deaths in cohort (Range)	214	214	216
	(134 - 309)	(134 - 309)	(134 - 309)
Total number of surgeries with UBT (Range)	4,615	2,817	9,411
	(1,223 - 12,545)	(1,223 - 11,395)	(1,223 - 15,615)
Total number of ICU admissions with UBT (Range)	4,932	3,594	8,500
	(3249 - 8,426)	(3249 - 7,741)	(3249 - 10,253)

ESM-Every Second Matters, UBT – Uterine Balloon Tamponade, INR – Indian National Rupee, USD – United

### Cost-effectiveness

Our model estimates use of condom-UBT in an atonic PPH case to be associated with 0.22 DALYs lost per patient. Using ESM-UBT as compared to condom-UBT incrementally averts 0.03 DALY per patient. Similarly, using Bakri-UBT is associated with 0.296 DALYs i.e. an additional 0.081 DALYs lost per patient using Bakri-UBT as compared to condom-UBT. An estimated 4,615 surgeries and 214 deaths are estimated to occur in an annual cohort of medically uncontrolled atonic PPH cases managed with condom-UBT device. Similarly, ESM-UBT use is estimated to be associated with 2,817 surgeries and 214 deaths. Bakri-UBT use is estimated to result in 9,411 surgeries and 216 deaths if used for atonic PPH management.

ESM-UBT versus condom-UBT in atonic PPH management has an ICER value of INR -2,412 per DALY averted i.e. an incremental cost-saving of INR 2,412 (USD 37) occurs with an incremental DALY averted by using ESM-UBT instead of the currently recommended condom-UBT intervention. At base-case, ESM-UBT is thus cost-saving as compared to condom-UBT. Similarly, Bakri-UBT versus condom-UBT has an ICER value of INR -126,219 per DALY averted, in this case however an incremental cost of INR 126,219 (USD 1,957) is incurred and one less DALY is averted by using Bakri-UBT instead of the recommended condom-UBT choice. Therefore, at base-case, Bakri-UBT is not cost-effective as compared to condom-UBT. Table 3 presents cost-effectiveness results for all three UBT devices used in the model.

**Table 3:** Incremental costs, consequences and cost-effectiveness of UBT intervention in atonic PPH management.

	ESM-UBT versus condom-UBT	Bakri-UBT versus condom-UBT
**Societal perspective (Per patient)**		
Incremental costs in INR (USD)	-73 (-1.15)	10,224 (158.5)
DALYs averted	0.030	-0.081
Incremental cost per DALY averted in INR (USD)	-2,412 (-37.39)	-126,219 (-1,957)
**Health system perspective (Per patient)**		
Incremental costs in INR (USD)	-73 (-1.15)	10,222 (158.5)
DALYs averted	0.030	-0.081
Incremental cost per DALY averted in INR (USD)	-2,414 (-37.79)	-126,201 (-1,957)

Societal net monetary benefit was estimated for UBT intervention comparisons to simplify the interpretation for ICER (DALY) values as seen with model results. At a WTP threshold of INR 24,211 (USD 375), ESM-UBT yields a base-case positive incremental NMB value of INR 809 (USD 13) per patient as compared to condom-UBT, suggesting ESM-UBT to be a cost-effective intervention in the NMB framework. Bakri-UBT yields a negative base-case incremental NMB value of INR -12,185 (USD -189) per patient as compared to condom-UBT, suggesting that Bakri-UBT is not cost-effective as compared to condom-UBT.

### Sensitivity analysis

**Fig 2:**
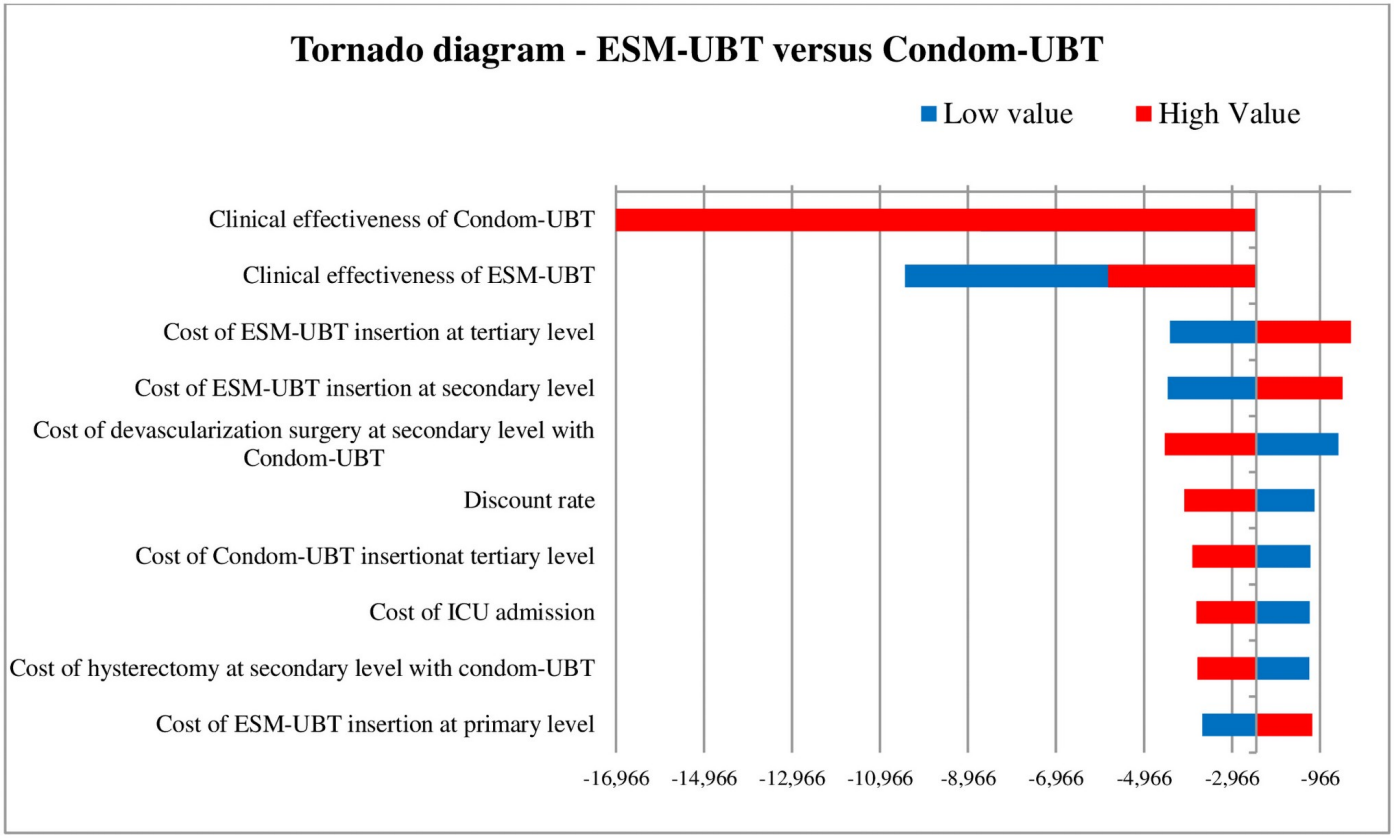
Tornado diagram showing one-way sensitivity analysis results for ESM-UBT versus condom-UBT comparison. As clinical effectiveness of condom-UBT increases, ESM-UBT is not cost-effective (ESM-UBT more expensive, less effective) as compared to condom-UBT. As clinical effectiveness of condom-UBT decreases (masked), ESM-UBT is cost-saving (ESM-UBT is less expensive, more effective) as compared to condom-UBT. Similarly, for ESM-UBT clinical effectiveness parameter, as clinical effectiveness of ESM-UBT increases, ESM-UBT is cost-saving (ESM-UBT less expensive, more effective) as compared to condom-UBT. As effectiveness of ESM-UBT decreases, ESM-UBT is not cost-effective (ESM-UBT more expensive, less effective) as compared to condom-UBT.

**Fig 3:**
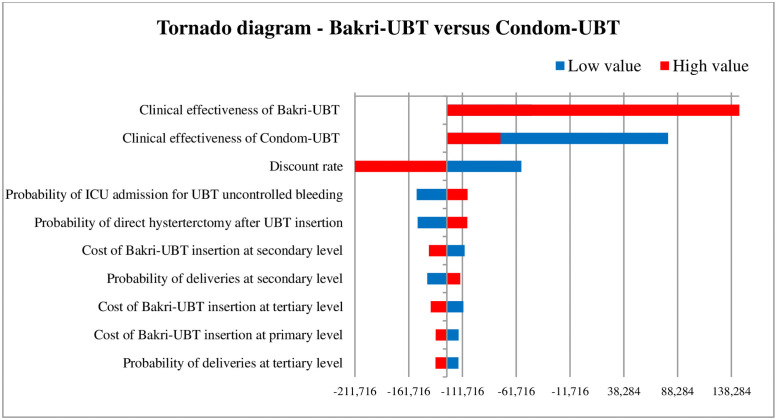
Tornado diagram showing one-way sensitivity analysis results for Bakri-UBT versus condom-UBT comparison. As clinical effectiveness of Bakri-UBT increases, Bakri-UBT is still not cost-effective (Bakri-UBT is more expensive, more effective) at the willingness to pay threshold of INR 24,211. As effectiveness of Bakri-UBT decreases (masked), Bakri-UBT is not cost-effective (Bakri-UBT is more expensive, less effective) as compared to condom-UBT. Similarly, for condom-UBT clinical effectiveness parameter, as condom-UBT effectiveness increases, Bakri-UBT is not cost-effective (Bakri-UBT is more expensive, less effective) as compared to condom-UBT. As condom-UBT effectiveness decreases, Bakri-UBT is still not cost-effective (Bakri-UBT is more expensive, more effective) at the given WTP threshold.

**Fig 4:**
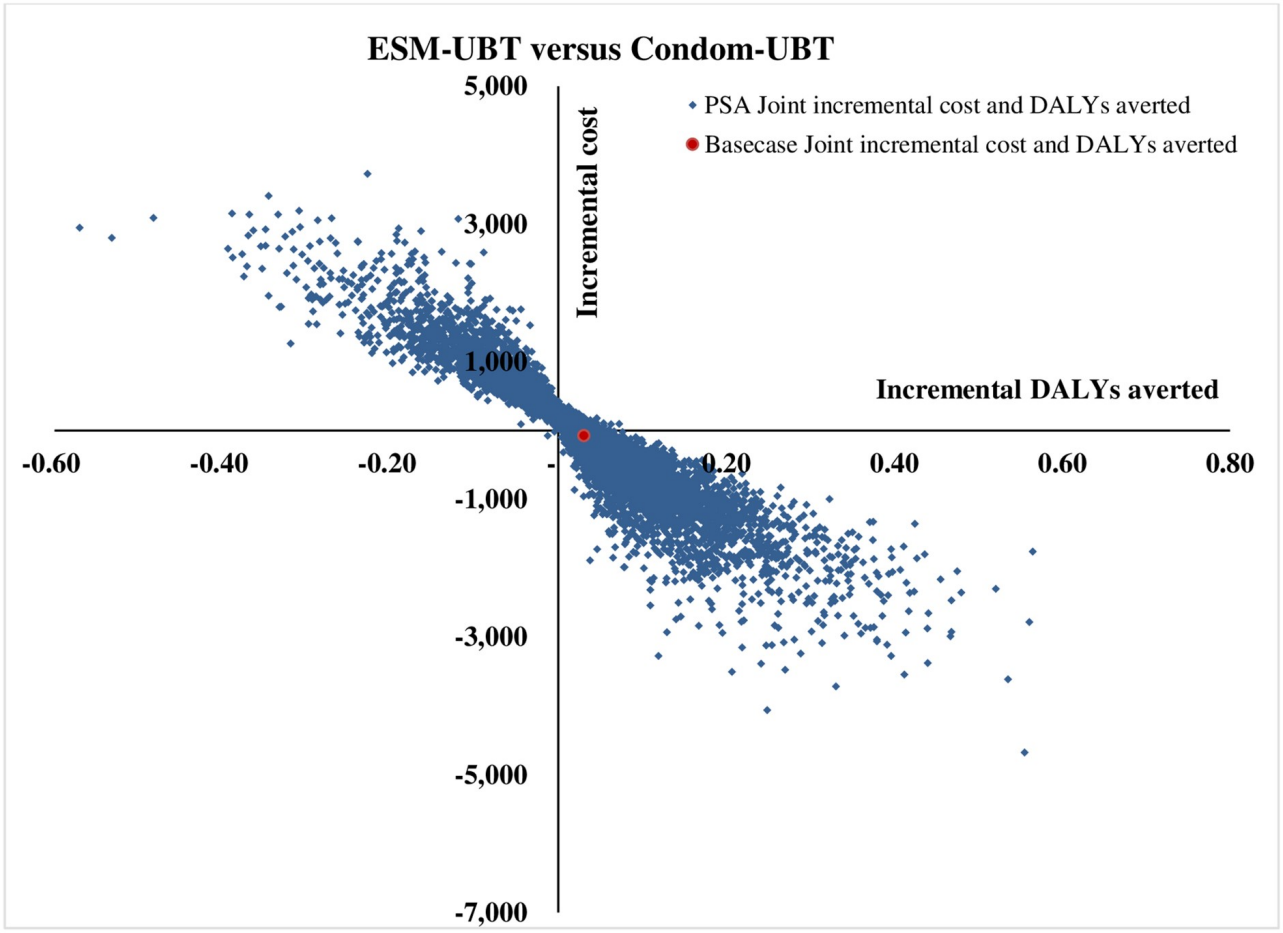
Probabilistic sensitivity analysis results with 10,000 Monte Carlo simulations for ESM-UBT versus condom-UBT comparison.

**Fig 5:**
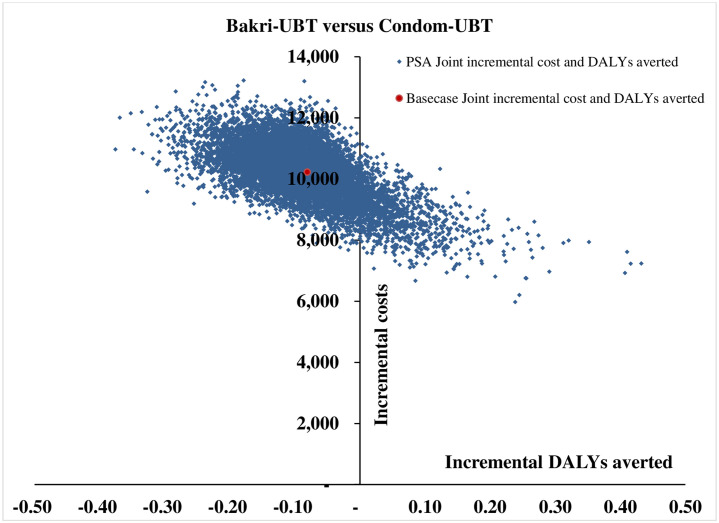
Probabilistic sensitivity analysis results with 10,000 Monte Carlo simulations for Bakri-UBT versus condom-UBT comparison.

The tornado diagram as shown in Fig 2 depicts the ten most-sensitive parameters influencing ICER (DALY averted) value for ESM-UBT versus condom-UBT comparison. Clinical effectiveness of condom-UBT, clinical effectiveness of ESM-UBT and cost of ESM-UBT insertion at tertiary facility are the three most influential parameters for ESM-UBT versus condom-UBT comparison. Similarly, for Bakri-UBT versus condom-UBT as shown in Fig 3, clinical effectiveness of Bakri-UBT, clinical effectiveness of condom-UBT and discount rate for outcomes are the three most influential parameters affecting ICER (DALY averted) values. The 10,000 Monte Carlo simulations of probabilistic sensitivity analysis for ESM-UBT versus Condom-UBT as seen in Fig 4, suggests a degree of uncertainty around base-case result with 63.5% of the simulations at the given WTP threshold turning cost-effective (52% dominant). For Bakri-UBT versus Condom-UBT comparison as seen in Fig 5, only 0.1% iterations were cost-effective at base-case WTP threshold (89% dominated).

## Discussion

This analysis presents new cost-effectiveness evidence regarding the choice of UBT devices available for management of atonic PPH complication in Indian public health settings. Globally, studies have assessed cost-effectiveness of various prophylactic or medical treatment options, estimated health system costs and modelled health outcomes for PPH management. A recent Indian study predicted that enhanced PPH care with non-surgical measures such as UBT intervention is cost-effective and life-saving [61]. Similarly, a Kenyan study from the year 2017 found ESM-UBT device to be highly cost-effective for severe PPH management in Kenyan health facility settings. Our study has analyzed and compared the choices of available UBT devices for atonic PPH management in Indian public health facilities using the economic evaluation approach.

Our base-case result suggests ESM-UBT for available limited clinical evidence to be associated with marginally lower costs, fewer DALYs, surgeries, deaths and an incremental positive net monetary benefit as compared to condom-UBT. The outcomes although favor ESM-UBT at base-case, are only marginal and absolute differences in societal costs, health outcomes and net benefits are minimal as compared to condom-UBT. The ICER value at base-case suggests ESM-UBT to be a cost-saving strategy. For this result, the three-time higher ESM-UBT device price as compared to condom-UBT is offset by fewer subsequent surgeries, associated deaths and thus a marginally lower societal expenditure with favorable health outcomes. Base-case comparison between Bakri-UBT and condom-UBT in our study suggests Bakri-UBT to be associated with significantly higher societal costs, higher DALYs lost, higher number of deaths, surgeries and a negative incremental monetary benefit as compared to condom-UBT. The associated ICER value indicates Bakri-UBT to be a less favorable intervention as compared to condom-UBT for India.

**Fig 6:**
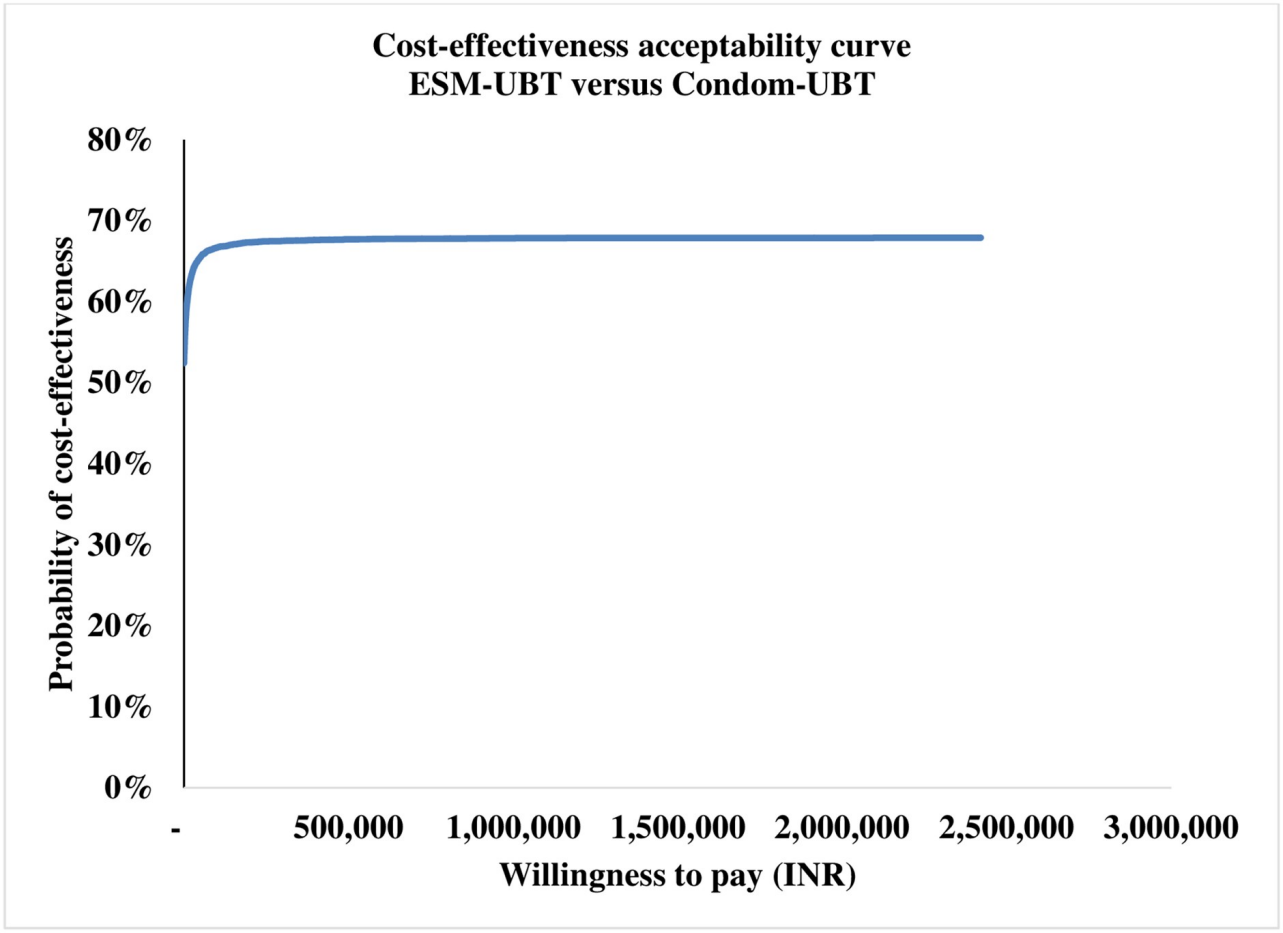
Cost-effectiveness acceptability curve for ESM-UBT versus condom-UBT comparison.

**Fig 7:**
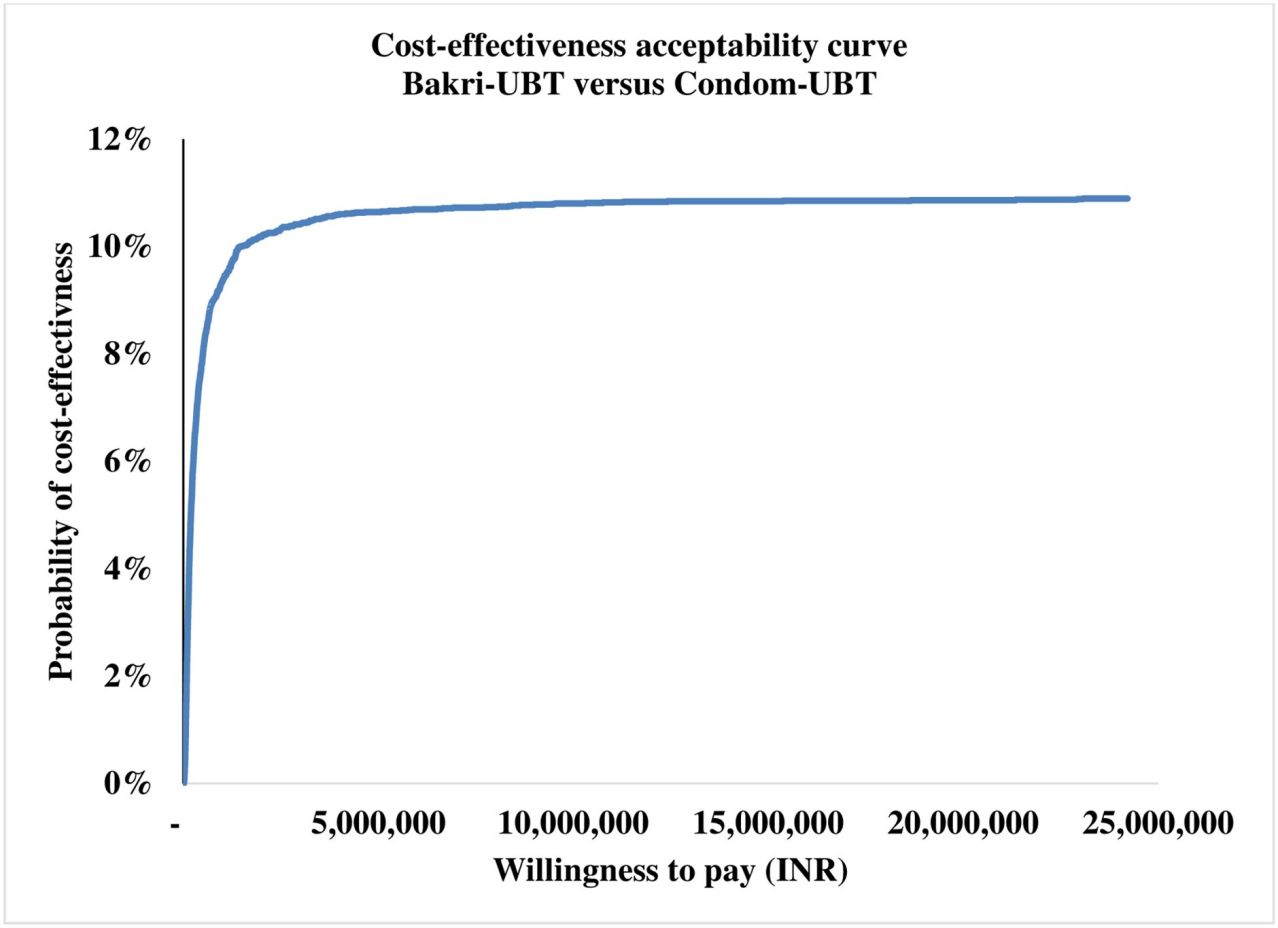
Cost-effectiveness acceptability curve for ESM-UBT versus condom-UBT comparison.

At WTP of INR 24,211, ESM-UBT was cost-effective in 63.5% Monte Carlo simulations as shown in Fig 6. With an increase in WTP to the WHO and India recommended one-time GDP per capita (INR 127,816), ESM-UBT turns cost-effective in 67% simulations, plateauing to a maximum of 68% for 3 time GDP per capita or higher thresholds. Similarly, PSA for Bakri-UBT versus Condom-UBT device comparison indicated only 0.1% i.e. six out of 10,000 Monte Carlo simulations to be cost-effective for Bakri-UBT intervention. For WHO and India recommended threshold of one-time GDP per capita, only 3% simulations turned cost-effective, plateauing at a maximum of 11% for extremely high thresholds as shown in Fig 7.

The ceiling 68% probability of ESM-UBT intervention being-cost effective in PSA analysis indicates a degree of uncertainty around model results. This uncertainty can be attributed to limitations associated with quality and nature of existing available clinical effectiveness evidence, importance of effectiveness parameter as reflected by OWSA results and non-specificity of input parameters used for individual UBT type. For the base-case net incremental monetary benefit of INR 809 per case with ESM-UBT, a mean incremental NMB value of INR 842 [95% CI 783 - 902] (USD 13 [95% CI 12 - 14]) per patient was computed using the 10,000 PSA simulations. Given the decision-making error probability and limited evidence available especially for ESM-UBT context, we undertook an expected value of information analysis. Total expected value of perfect information (EVPI) for population was determined to quantify the monetary need for evidence generation that reduces decision uncertainty. For this calculation, an undiscounted one-time population cohort was used. The estimated value of population EVPI for ESM-UBT versus condom-UBT was INR 14,323,056 (USD 222,063) for an annual cohort. Given the sensitive nature of model results to clinical effectiveness parameter, the EVPI result can be used by decision makers to assess research prioritization for generation of clinical evidence. Robust comparative clinical effectiveness evidence especially for ESM-UBT versus condom-UBT comparison in the Indian context can be considered to improve and determine net incremental associated benefits to the society. For uncertainty across sensitivity analysis results and marginal differences in associated costs and health outcomes, a cost-saving result for ESM-UBT with existing evidence must be considered with caution and adoption of ESM-UBT in the Indian public heath settings for atonic PPH management instead of the currently recommended condom-UBT device must be made after due deliberation.

Bakri-UBT device with significantly higher societal costs and lesser favorable health outcomes was dominated by condom-UBT across base-case and PSA with a ceiling 11% probability of Bakri-UBT being cost-effective. The base-case net incremental monetary benefit for Bakri-UBT was INR -12,184 with a mean value of INR -12,299 [95% CI -12,349, -12,249] (USD -191 [95% CI -191, -190]). Condom-UBT device remains a more cost-effective alternative as compared to Bakri-UBT device in Indian health settings across analysis. However, in a scenario with Bakri-UBT device compared against no UBT intervention at all for atonic PPH management, although Bakri-UBT device has high associated societal costs, relative health outcomes and incremental cost-effectiveness ratios are still favorable for Bakri-UBT, suggesting UBT intervention to be a favorable alternative as compared to not providing UBT intervention at all for medically uncontrolled cases.

This study has its strengths and limitations. To our knowledge, this is the first such study that has presented cost-effectiveness evidence by comparing available UBT devices for atonic PPH management. We have analyzed cost-effectiveness of UBT devices available in the Indian context using India specific primary costs, epidemiologic and clinical effectiveness data to the available extent. A detailed sensitivity analysis was undertaken in this study to address uncertainties and assumptions used in the model. For the reported uncertainty in our results, we have presented the potential monetary value associated with generation of evidence by undertaking further research to reduce existing uncertainties and further improving robustness in decision making.

The study limitations include use of clinical effectiveness input parameter based out of limited robust evidence currently available for all UBT devices, especially for ESM-UBT which is based on findings from three case-series studies that largely assess survival outcomes in African health settings. Unavailability of clinical effect estimates from a systematic robust comparison of clinical effectiveness between UBT devices over identical outcome measures is an important limitation of the study results. Secondly, the study uses disability weights that non-specifically measures disease burden rather than assessing specific health state outcomes. Though QALY estimation for decision making as recommended in India may have been a more accurate measure, we chose to use condition specific DALY outcome instead of using proxy utility weights from other contextual settings for QALY estimation. Thirdly, the model does not consider potential costs and consequences of a complication like anemia as it is a common morbidity prevalent among Indian women throughout pregnancy and post-partum period [62]. However, given the research question at hand, decision model for given intervention was considered adequate to assist decision-makers. We relied on PPH non-specific out-of-pocket expenditure for childbirth to estimate disaggregated societal costs. Additionally, indirect costs like wage loss estimation or productivity loss were not included. Availability of these PPH specific costs may further improve result accuracy. Finally, the study bases its health system cost findings from sample facilities of one contextual setting within India. Though sensitivity analysis plausibly varies health system and OOPE costs, the representativeness of these costs for all India settings may still be limited.

## Conclusion

This study demonstrates that condom-UBT device as recommended for atonic PPH management in India offers better value as compared to Bakri-UBT in Indian public health settings. Although ESM-UBT presents as a cost-saving alternative, decision making between low-cost ESM-UBT and condom-UBT needs further evaluation as differences in costs and health outcomes are marginal, there is uncertainty associated with cost-effectiveness results and clinical evidence available for ESM-UBT is limited at present. Going ahead, high-quality comparative clinical effectiveness evidence is needed to reduce decision-making uncertainty around the choice of UBT device. Future research can also focus on generating India specific health utility scores for PPH, qualitative aspects such as ease of use and provider preferences for UBT devices. Our study results can assist policy makers in prioritizing interventions for atonic PPH management in India. Supply of an affordable and acceptable UBT device particularly needs to be complemented by health system preparedness in the form of skilled workforce and robust referral systems to tackle the PPH emergencies.

## Supporting information

S1 Table Details of studies included in literature review of the three UBT devices. (DOCX)

S1 Appendix Checklists for study validation and reporting. (DOC)
