# Supplementary material for: Cost-effectiveness of uterine balloon tamponade devices in managing atonic post-partum hemorrhage at public health facilities in India
Source: PLoS One. 2021 Aug 18;16(8):e0256271. doi: 10.1371/journal.pone.0256271 (PMC8372914; doi:10.1371/journal.pone.0256271)
Supplement: S1 Table — (DOCX) [file pone.0256271.s001.docx]

**S1 Table. Details of studies included in literature review of the three UBT devices**

| **Authors** | **Study design** | **PPH success rate** | **Atonic PPH success rate** | **Reference** |
| --- | --- | --- | --- | --- |
| **Condom-UBT** | | | | |
| Darwish et al. | RCT | 28/33 (84.8) | 28/33 (84.8) | [1] |
| Tindell et al. | Systematic Review | 186/193 (96.4) | NR | [2] |
| Santhanam et al. | Prospective | 59/61 (96.7) | 59/61 (96.7) | [3] |
| Rathore et al. | Prospective | 17/18 (94.4) | NR | [4] |
| Aderoba et al. | Prospective | 203/229 (88.6) | 193/214 (90.2) | [5] |
| Mishra et al. | Prospective | 59/60 (98.3) | NR | [6] |
| Kandeel et al. | Prospective | 48/50 (96.0) | 28/28 (100) | [7] |
| Anger et al. | RCT | 56/64 (87.5) | NR | [8] |
| Dumont et al. | RCT | 48/57 (84.2) | NR | [9] |
| Lohano et al. | Prospective | 126/139 (90.6) | 126/139 (90.6) | [10] |
| Hasabe et al. | Prospective | 34/36 (94.4) | NR | [11] |
| Yadav et al. | Prospective | 117/122 (95.9) | 117/122 (95.9) | [12] |
| **Bakri-UBT** | | | | |
| Darwish et al. | RCT | 30/33 (90.9) | 30/33 (90.9) | [1] |
| Revert et al. | Prospective | 188/226 (83.2) | 155/183 (84.7) | [13] |
| Brown et al. | Prospective | 55/58 (94.8) | 52/55 (94.5) | [14] |
| Vintejoux et al. | Retrospective | 25/36 (69.4) | 25/36 (69.4) | [15] |
| Guo et al. | Retrospective | 288/305 (94.4) | 131/142 (92.3) | [16] |
| Mathur et al. | Retrospective | 40/49 (81.6) | 14/17 (82.4) | [17] |
| Wang et al. | Prospective | 373/407 (91.6) | 373/407 (91.6) | [18] |
| Alkis et al. | Retrospective | 43/47 (91.5) | NR | [19] |
| Kaya et al. | Prospective | 34/45 (75.6) | 27/34 (79.4) | [20] |
| Laas et al. | Before and after | 37/43 (86) | 37/43 (86) | [21] |
| Olsen et al. | Retrospective | 25/37 (67.6) | 17/24 (70.8) | [22] |
| Kong et al. | Retrospective | 59/81 (72.8) | 37/59 (62.7) | [23] |
| Cetin et al. | Retrospective | 29/39 (74.4) | 29/39 (74.4) | [24] |
| Gauchotte et al. | Before and after | 35/38 (92.1) | NR | [25] |
| Grange et al. | Retrospective | 80/108 (74.1) | 26/39 (66.7) | [26] |
| Kadioglu et al. | Retrospective | 42/50 (84) | NR | [27] |
| Martin et al. | Retrospective | 32/49 (65.3) | 28/42 (66.7) | [28] |
| Ogoyama et al. | Retrospective | 66/71 (93) | 31/32 (96.9) | [29] |
| Son et al. | Retrospective | 239/306 (78.1) | 190/241 (78.8) | [30] |
| **ESM-UBT** | | | | |
| Ramanathan et al | Prospective/ Retrospective case series | 189/201  (94) * | NR | [31] |
| Burke et al. | Prospective case series | 190/201  (94.5) * | NR | [32] |
| Burke et al. | Prospective case series | 298/306  (97.4) * | 298/306 (97.4) | [33] |

REFERENCES

1. Darwish AM, Abdallah MM, Shaaban OM, Ali MK, Khalaf M, Sabra AMA. Bakri balloon versus condom-loaded Foley’s catheter for treatment of atonic postpartum hemorrhage secondary to vaginal delivery: a randomized controlled trial. J Matern Neonatal Med. 2018;31: 747–753. doi:10.1080/14767058.2017.1297407

2. Tindell K, Garfinkel R, Abu-Haydar E, Ahn R, Burke TF, Conn K, et al. Uterine balloon tamponade for the treatment of postpartum haemorrhage in resource-poor settings: A systematic review. BJOG: An International Journal of Obstetrics and Gynaecology. 2013. pp. 5–14. doi:10.1111/j.1471-0528.2012.03454.x

3. Santhanam R, Viswanathan RM, V. P. Condom tamponade in the management of atonic postpartum hemorrhage. Int J Reprod Contraception, Obstet Gynecol. 2018;7: 2276. doi:10.18203/2320-1770.ijrcog20182335

4. Rathore AM, Gupta S, Manaktala U, Gupta S, Dubey C, Khan M. Uterine tamponade using condom catheter balloon in the management of non-traumatic postpartum hemorrhage. J Obstet Gynaecol Res. 2012;38: 1162–1167. doi:10.1111/j.1447-0756.2011.01843.x

5. Aderoba A, Olagbuji B, Akintan A, Oyeneyin O, Owa O, Osaikhuwuomwan J. Condom-catheter tamponade for the treatment of postpartum haemorrhage and factors associated with success: a prospective observational study. BJOG An Int J Obstet Gynaecol. 2017;124: 1764–1771. doi:10.1111/1471-0528.14361

6. Mishra N, Gulabani K, Agrawal S, Shrivastava C. Efficacy and Feasibility of Chhattisgarh Balloon and Conventional Condom Balloon Tamponade: A 2-Year Prospective Study. J Obstet Gynecol India. 2019;69: 133–141. doi:10.1007/s13224-018-1185-6

7. Kandeel M, Sanad Z, Ellakwa H, El Halaby A, Rezk M, Saif I. Management of postpartum hemorrhage with intrauterine balloon tamponade using a condom catheter in an Egyptian setting. Int J Gynecol Obstet. 2016;135: 272–275. doi:10.1016/j.ijgo.2016.06.018

8. Anger HA, Dabash R, Durocher J, Hassanein N, Ononge S, Frye LJ, et al. The effectiveness and safety of introducing condom-catheter uterine balloon tamponade for postpartum haemorrhage at secondary level hospitals in Uganda, Egypt and Senegal: a stepped wedge, cluster-randomised trial. BJOG An Int J Obstet Gynaecol. 2019;126: 1612–1621. doi:10.1111/1471-0528.15903

9. Dumont A, Bodin C, Hounkpatin B, Popowski T, Traoré M, Perrin R, et al. Uterine balloon tamponade as an adjunct to misoprostol for the treatment of uncontrolled postpartum haemorrhage: A randomised controlled trial in Benin and Mali. BMJ Open. 2017;7: 1–9. doi:10.1136/bmjopen-2017-016590

10. Lohano R, Haq G, Kazi S, Sheikh S. Intrauterine balloon tamponade for the control of postpartum haemorrhage. J Pak Med Assoc. 2016;66: 22–26.

11. Hasabe R, Gupta K, Rathode P. Use of Condom Tamponade to Manage Massive Obstetric Hemorrhage at a Tertiary Center in Rajasthan. J Obstet Gynecol India. 2016;66: 88–93. doi:10.1007/s13224-015-0790-x

12. Yadav S, Malhotra A. A prospective randomized comparative study of Misoprostol and balloon tamponade using condom catheter to prevent postpartum hemorrhage at M. Y. H., Indore, India in vaginal delivered patients. Int J Reprod Contraception, Obstet Gynecol. 2019;8: 591. doi:10.18203/2320-1770.ijrcog20190290

13. Revert M, Cottenet J, Raynal P, Cibot E, Quantin C, Rozenberg P. Intrauterine balloon tamponade for management of severe postpartum haemorrhage in a perinatal network: a prospective cohort study. BJOG An Int J Obstet Gynaecol. 2017;124: 1255–1262. doi:10.1111/1471-0528.14382

14. Brown H, Okeyo S, Mabeya H, Wilkinson J, Schmitt J. The Bakri tamponade balloon as an adjunct treatment for refractory postpartum hemorrhage. Int J Gynecol Obstet. 2016;135: 276–280. doi:10.1016/j.ijgo.2016.06.021

15. Vintejoux E, Ulrich D, Mousty E, Masia F, Marès P, De Tayrac R, et al. Success factors for Bakri^TM^ balloon usage secondary to uterine atony: A retrospective, multicentre study. Aust New Zeal J Obstet Gynaecol. 2015;55: 572–577. doi:10.1111/ajo.12376

16. Guo Y, Hua R, Bian S, Xie X, Ma J, Cai Y, et al. Intrauterine Bakri Balloon and Vaginal Tamponade Combined with Abdominal Compression for the Management of Postpartum Hemorrhage. J Obstet Gynaecol Canada. 2018;40: 561–565. doi:10.1016/j.jogc.2017.08.035

17. Mathur M, Ng QJ, Tagore S. Use of Bakri balloon tamponade (BBT) for conservative management of postpartum haemorrhage: a tertiary referral centre case series. J Obstet Gynaecol (Lahore). 2018;38: 66–70. doi:10.1080/01443615.2017.1328671

18. Wang D, Xu S, Qiu X, Zhu C, Li Z, Wang Z, et al. Early usage of Bakri postpartum balloon in the management of postpartum hemorrhage: A large prospective, observational multicenter clinical study in South China. J Perinat Med. 2018;46: 649–656. doi:10.1515/jpm-2017-0249

19. Alkiş I, Karaman E, Han A, Ark HC, Büyükkaya B. The fertility sparing management of postpartum hemorrhage: A series of 47 cases of Bakri balloon tamponade. Taiwan J Obstet Gynecol. 2015;54: 232–235. doi:10.1016/j.tjog.2014.03.009

20. Kaya B, Tuten A, Daglar K, Misirlioglu M, Polat M, Yildirim Y, et al. Balloon tamponade for the management of postpartum uterine hemorrhage. J Perinat Med. 2014;42: 745–753. doi:10.1515/jpm-2013-0336

21. Laas E, Bui C, Popowski T, Mbaku OM, Rozenberg P. Trends in the rate of invasive procedures after the addition of the intrauterine tamponade test to a protocol for management of severe postpartum hemorrhage. Am J Obstet Gynecol. 2012;207: 281.e1-281.e7. doi:10.1016/j.ajog.2012.08.028

22. Olsen R, Reisner DP, Benedetti TJ, Dunsmoor-Su RF. Bakri balloon effectiveness for postpartum hemorrhage: A “real world experience.” J Matern Neonatal Med. 2013;26: 1720–1723. doi:10.3109/14767058.2013.796354

23. Kong CW, To WW. Prognostic factors for the use of intrauterine balloon tamponade in the management of severe postpartum hemorrhage. Int J Gynecol Obstet. 2018;142: 48–53. doi:10.1002/ijgo.12498

24. Çetin BA, Aydogan Mathyk B, Atis Aydin A, Koroglu N, Yalcin Bahat P, Temel Yuksel I, et al. Comparing success rates of the Hayman compression suture and the Bakri balloon tamponade. J Matern Neonatal Med. 2019;32: 3034–3038. doi:10.1080/14767058.2018.1455184

25. Gauchotte E, De La Torre M, Perdriolle-Galet E, Lamy C, Gauchotte G, Morel O. Impact of uterine balloon tamponade on the use of invasive procedures in severe postpartum hemorrhage. Acta Obstet Gynecol Scand. 2017;96: 877–882. doi:10.1111/aogs.13130

26. Grange J, Chatellier M, Chevé MT, Paumier A, Launay-Bourillon C, Legendre G, et al. Predictors of failed intrauterine balloon tamponade for persistent postpartum hemorrhage after vaginal delivery. PLoS One. 2018;13: 1–11. doi:10.1371/journal.pone.0206663

27. Kadioglu BG, Tanriverdi EC, Aksoy AN. Balloon Tamponade in the Management of Postpartum Hemorrhage: Three Years of Experience in a Single Center. Open J Obstet Gynecol. 2016;06: 698–704. doi:10.4236/ojog.2016.612087

28. Martin E, Legendre G, Bouet PE, Cheve MT, Multon O, Sentilhes L. Maternal outcomes after uterine balloon tamponade for postpartum hemorrhage. Acta Obstet Gynecol Scand. 2015;94: 399–404. doi:10.1111/aogs.12591

29. Ogoyama M, Takahashi H, Usui R, Baba Y, Suzuki H, Ohkuchi A, et al. Hemostatic effect of intrauterine balloon for postpartum hemorrhage with special reference to concomitant use of “holding the cervix” procedure (Matsubara). Eur J Obstet Gynecol Reprod Biol. 2017;210: 281–285. doi:10.1016/j.ejogrb.2017.01.012

30. Son M, Einerson BD, Schneider P, Fields IC, Grobman WA, Miller ES. Is There an Association between Indication for Intrauterine Balloon Tamponade and Balloon Failure? Am J Perinatol. 2017;34: 164–168. doi:10.1055/s-0036-1585084

31. Ramanathan A, Eckardt MJ, Nelson BD, Guha M, Oguttu M, Altawil Z, et al. Safety of a condom uterine balloon tamponade (ESM-UBT) device for uncontrolled primary postpartum hemorrhage among facilities in Kenya and Sierra Leone. BMC Pregnancy Childbirth. 2018;18: 1–7. doi:10.1186/s12884-018-1808-z

32. Burke TF, Ahn R, Nelson BD, Hines R, Kamara J, Oguttu M, et al. A postpartum haemorrhage package with condom uterine balloon tamponade: a prospective multi-centre case series in Kenya, Sierra Leone, Senegal, and Nepal. BJOG An Int J Obstet Gynaecol. 2016;123: 1532–1540. doi:10.1111/1471-0528.13550

33. Burke TF, Danso-Bamfo S, Guha M, Oguttu M, Tarimo V, Nelson BD. Shock progression and survival after use of a condom uterine balloon tamponade package in women with uncontrolled postpartum hemorrhage. Int J Gynecol Obstet. 2017;139: 34–38. doi:10.1002/ijgo.12251
