# Supplementary material for: Cost-effectiveness of uterine balloon tamponade devices in managing atonic post-partum hemorrhage at public health facilities in India
Source: PLoS One. 2021 Aug 18;16(8):e0256271. doi: 10.1371/journal.pone.0256271 (PMC8372914; doi:10.1371/journal.pone.0256271)
Supplement: S1 Appendix — (DOC) [file pone.0256271.s002.DOC]

**S1 Appendix**

**CHECKLISTS FOR STUDY VALIDATION AND REPORTING**

**Part-1**

**CHEERS STATEMENT**

CHEERS checklist—Items to include when reporting economic evaluations of health interventions

| **Section/item** | **Item No** | **Recommendation** | **Reported on page No/ line No** |
| --- | --- | --- | --- |
| **Title and abstract** | | | |
| Title | 1 | Identify the study as an economic evaluation or use more specific terms such as “cost-effectiveness analysis”, and describe the interventions compared. | Page no. 01, Line no. 01 to 02 |
| Abstract | 2 | Provide a structured summary of objectives, perspective, setting, methods (including study design and inputs), results (including base case and uncertainty analyses), and conclusions. | Page no. 03, Line no. 53 to 78 |
| **Introduction** | | | |
| Background and objectives | 3 | Provide an explicit statement of the broader context for the study. | Page no. 05, Line no. 121 to 126  Page no. 06, Line no. 133 to 137 |
|  |  | Present the study question and its relevance for health policy or practice decisions. | Page no. 06, Line no. 133 to 137 |
| **Methods** | | | |
| Target population and subgroups | 4 | Describe characteristics of the base case population and subgroups analysed, including why they were chosen. | Page no. 06, Line no. 152 to 154 |
| Setting and location | 5 | State relevant aspects of the system(s) in which the decision(s) need(s) to be made. | Page no. 07 to 08. Line no. 176 to 190 |
| Study perspective | 6 | Describe the perspective of the study and relate this to the costs being evaluated. | Page no. 06, Line no. 146 to 147 |
| Comparators | 7 | Describe the interventions or strategies being compared and state why they were chosen. | Page no. 06, Line no. 142 to 144 |
| Time horizon | 8 | State the time horizon(s) over which costs and consequences are being evaluated and say why appropriate. | Page no. 06 to 07, Line no. 155 to 160 |
| Discount rate | 9 | Report the choice of discount rate(s) used for costs and outcomes and say why appropriate. | Page no. 07, Line no. 160 to 163 |
| Choice of health outcomes | 10 | Describe what outcomes were used as the measure(s) of benefit in the evaluation and their relevance for the type of analysis performed. | Page no. 06, Line no. 148 to 149  Page no. 13, Line no. 261 to 265 |
| Measurement of effectiveness | 11a | *Single study-based estimates:*Describe fully the design features of the single effectiveness study and why the single study was a sufficient source of clinical effectiveness data. | Not applicable |
|  | 11b | *Synthesis-based estimates*: Describe fully the methods used for identification of included studies and synthesis of clinical effectiveness data. | Page no.08 to 09, Line no. 200 to 226  Table 1 |
| Measurement and valuation of preference based outcomes | 12 | If applicable, describe the population and methods used to elicit preferences for outcomes. | Not applicable |
| Estimating resources and costs | 13a | *Single study-based economic evaluation:* Describe approaches used to estimate resource use associated with the alternative interventions. Describe primary or secondary research methods for valuing each resource item in terms of its unit cost. Describe any adjustments made to approximate to opportunity costs. | Not applicable |
|  | 13b | *Model-based economic evaluation:*Describe approaches and data sources used to estimate resource use associated with model health states. Describe primary or secondary research methods for valuing each resource item in terms of its unit cost. Describe any adjustments made to approximate to opportunity costs. | Page no. 12 to 13, Line no. 232 to 259  Table 1 |
| Currency, price date, and conversion | 14 | Report the dates of the estimated resource quantities and unit costs. Describe methods for adjusting estimated unit costs to the year of reported costs if necessary. Describe methods for converting costs into a common currency base and the exchange rate. | Page no. 12 to 13, Line no. 232 to 259 |
| Choice of model | 15 | Describe and give reasons for the specific type of decision-analytical model used. Providing a figure to show model structure is strongly recommended. | Page no. 06, Line no. 145 to 146  Figure 1 |
| Assumptions | 16 | Describe all structural or other assumptions underpinning the decision-analytical model. | Page no. 08, Line no. 191 to 197  Page no.13, Line no. 252 to 254 |
| Analytical methods | 17 | Describe all analytical methods supporting the evaluation. This could include methods for dealing with skewed, missing, or censored data; extrapolation methods; methods for pooling data; approaches to validate or make adjustments (such as half cycle corrections) to a model; and methods for handling population heterogeneity and uncertainty. | Page no. 14 to 19, Line no. 298 to 330  Table 1 |
| **Results** | | | |
| Study parameters | 18 | Report the values, ranges, references, and, if used, probability distributions for all parameters. Report reasons or sources for distributions used to represent uncertainty where appropriate. Providing a table to show the input values is strongly recommended. | Page no. 15, Line no. 308 to 326  Table 1 |
| Incremental costs and outcomes | 19 | For each intervention, report mean values for the main categories of estimated costs and outcomes of interest, as well as mean differences between the comparator groups. If applicable, report incremental cost-effectiveness ratios. | Page no. 16 to 17, Line no. 335 to 355  Table 2  Table 3 |
| Characterising uncertainty | 20a | *Single study-based economic evaluation:* Describe the effects of sampling uncertainty for the estimated incremental cost and incremental effectiveness parameters, together with the impact of methodological assumptions (such as discount rate, study perspective). | Not applicable |
|  | 20b | *Model-based economic evaluation:*Describe the effects on the results of uncertainty for all input parameters, and uncertainty related to the structure of the model and assumptions. | Page no. 18 to 19, Line no. 371 to 400  Figure 2  Figure 3  Figure 4  Figure 5 |
| Characterising heterogeneity | 21 | If applicable, report differences in costs, outcomes, or cost-effectiveness that can be explained by variations between subgroups of patients with different baseline characteristics or other observed variability in effects that are not reducible by more information. | Not applicable |
| **Discussion** | | | |
| Study findings, limitations, generalisability, and current knowledge | 22 | Summarise key study findings and describe how they support the conclusions reached. Discuss limitations and the generalisability of the findings and how the findings fit with current knowledge. | Page no. 19 to 22, Line no. 403 to 473 |
| **Other** | | | |
| Source of funding | 23 | Describe how the study was funded and the role of the funder in the identification, design, conduct, and reporting of the analysis. Describe other non-monetary sources of support. | Information provided via submission system |
| Conflicts of interest | 24 | Describe any potential for conflict of interest of study contributors in accordance with journal policy. In the absence of a journal policy, we recommend authors comply with International Committee of Medical Journal Editors recommendations. | Information provided via submission system |

For consistency, the CHEERS statement checklist format is based on the format of the CONSORT statement checklist

**Part-2**

**AdViSHE TOOL**

**Assessment of the Validation Status of Health-Economic decision models**

AdViSHE is a questionnaire that modellers can complete to report on the efforts performed to improve the validation status of their health-economic (HE) decision model. It is not intended to replace validation by model users but rather to inform the direction of validation efforts and to provide a baseline for replication of the results. In addition to using it after a model is finished, the modellers can use AdViSHE to guide validation efforts during the modelling process.

The modellers are asked to comment on the validation efforts performed while building the underlying HE decision model and afterwards. Many of the questions simply refer to the model documentation. AdViSHE is divided into five parts, each covering an aspect of validation:

- Part A: Validation of the conceptual model (2 questions)

- Part B: Input data validation (2 questions)

- Part C: Validation of the computerized model (4 questions)

- Part D: Operational validation (4 questions)

- Part E: Other validation techniques (1 question)

No final validation score is calculated, as the assessment of the answers and the overall validation effort is left to the model users. It is assumed that the model has been built according to prevailing modelling and reporting guidelines. Some questions may not be applicable to a particular model. If this is the case, the model builder should take the opt-out option and provide a justification of why this item is not deemed applicable.

**Table:** Author responses to the Advishe Model validation tool

|  | **AdViSHE tool** | **Model validation for Uterine Balloon Tamponade devices in atonic PPH management** |
| --- | --- | --- |
|  | Part A: Validation of the conceptual model (2 questions)  Part A discusses techniques for validating the conceptual model. A conceptual model describes the underlying system (e.g., progression of disease) using a mathematical, logical, verbal, or graphical representation. Please indicate where the conceptual model and its underlying assumptions are described and justified. | The conceptual model and schematic diagram of the decision analytic model is described in the manuscript along with the figure of the model (Figure 1) |
| A1 | Face validity testing (conceptual model): Have experts been asked to judge the appropriateness of the conceptual model?  If yes, please provide information on the following aspects:  - Who are these experts?  - What is your justification for considering them experts?  - To what extent do they agree that the conceptual model is appropriate?  If no, please indicate why not.  Aspects to judge include: appropriateness to represent the underlying clinical process/disease (disease stages, physiological processes, etc.); and appropriateness for economic evaluation (comparators, perspective, costs covered, etc.). | Experts validated the conceptual model.   1. The Technical Advisory Committee for Health Technology Assessment in India validated the conceptual model. This committee comprises of clinicians, public health professionals, health economists’ and other senior experts with vast experience in their respective fields.   After discussion, the experts agreed to a large extent that the conceptual model was acceptable. |
| A2 | Cross validity testing (conceptual model): Has this model been compared to other conceptual models found in the literature or clinical textbooks?  If yes, please indicate where this comparison is reported.  If no, please indicate why not. | Yes, we came across only one similar model from African setting. This study was reviewed during model conceptualization and comparative findings to the possible extent is reported in the introduction and discussion section of the manuscript. |
|  | Part B: Input data validation (2 questions)  Part B discusses techniques to validate the data serving as input in the model. These techniques are applicable to all types of models commonly used in Health economic modelling.  Please indicate where the description and justification of the following aspects are given:  - search strategy;  - data sources, including descriptive statistics;  - reasons for inclusion of these data sources;  - reasons for exclusion of other available data sources;  - assumptions that have been made to assign values to parameters for which no data was available;  - distributions and parameters to represent uncertainty;  - data adjustments: mathematical transformations (e.g., logarithms, squares); treatment of outliers; treatment of missing data; data synthesis (indirect treatment comparison, network meta-analysis); calibration; etc. | Details regarding search strategy; data sources, including descriptive statistics; distributions and parameters to represent uncertainty, assumptions that have been made to assign values to parameters for which no data was available, reasons for inclusion or exclusion of data sources is presented in the methods section of the manuscript. Treatment of outliers; treatment of missing data; data synthesis (indirect treatment comparison, network meta-analysis); calibration were not applicable to this sudy. |
| B1 | Face validity testing (input data): Have experts been asked to judge the appropriateness of the input data?  If yes, please provide information on the following aspects:  - Who are these experts?  - What is your justification for considering them experts?  - To what extent do they agree that appropriate data has been used?  If no, please indicate why not.  Aspects to judge may include but are not limited to: potential for bias; generalizability to the target population; availability of alternative data sources; any adjustments made to the data. | An expert was asked to validate the inputs for the model.   1. A senior health economist with more than five years of experience in health economic modelling having worked across various sectors in the Indian context validated the inputs in the model.   The expert agreed that most inputs used were appropriate. The expert discusssed and sought clarification for some of the input parameters used for health outcome measurement in the model. The effect of non-specific proxy utility parameters due to unavailability of India-specific utility weights was discussed and a consensus was achieved for all the input data used in the model. |
| B2 | Model fit testing: When input parameters are based on regression models, have statistical tests been performed?  If yes, please indicate where the description, the justification and the outcomes of these tests are reported.  If no, please indicate why not  Examples of regression models include but are not limited to: disease progression based on survival curves; risk profiles using regression analysis on a cohort; local cost estimates based on multi-level models; meta-regression; quality-of-life weights estimated using discrete choice analysis; mapping of disease-specific quality-of-life weights to utility values.  Examples of tests include but are not limited to: comparing model fit parameters (R2, AIC, BIC); comparing alternative model specifications (covariates, distributional assumptions); comparing alternative distributions for survival curves (Weibull, lognormal, logit); testing the numerical stability of the outcomes (sufficient number of iterations); testing the convergence of the regression model; visually testing model fit and/or regression residuals. | Input parameters were not based on regression models |
|  | Part C: Validation of the computerized model (4 questions)  Part C discusses various techniques for validating the model as it is implemented in a software program. If there are any differences between the conceptual model (Part A) and the final computerized model, please indicate where these differences are reported and justified. | The mathematical model is as per the conceptual model. |
| C1 | External review: Has the computerized model been examined by modelling experts?  If yes, please provide information on the following aspects:  - Who are these experts?  - What is your justification for considering them experts?  - Can these experts be qualified as independent?  - Please indicate where the results of this review are reported, including a discussion of any unresolved issues.  If no, please indicate why not.  Aspects to judge may include but are not limited to: absence of apparent bugs; logical code structure optimized for speed and accuracy; appropriate translation of the conceptual model. | An expert was asked to validate the computerized model for the model.  1) A senior health economist with more than five years of experience in health economic modelling having worked across various sectors in the Indian context validated the computerized model.  Yes, the expert is independent of our research. The result of model review was communicated personally and is not published anywhere. There were no unresolved issues after the model review. |
| C2 | Extreme value testing: Has the model been run for specific, extreme sets of parameter values in order to detect any coding errors?  If yes, please indicate where these tests and their outcomes are reported.  If no, please indicate why not.  Examples include but are not limited to: zero and extremely high (background) mortality; extremely beneficial, extremely detrimental, or no treatment effect; zero or extremely high treatment or healthcare costs. | Yes, extreme value testing has been done and has been reported in the discussion section of the manuscript. |
| C3 | Testing of traces: Have patients been tracked through the model to determine whether its logic is correct?  If yes, please indicate where these tests and their outcomes are reported.  If no, please indicate why not.  In cohort models, this would involve listing the number of patients in each disease stage at one, several, or all time points (e.g., Markov traces). In individual patient simulation models, this would involve following several patients throughout their natural disease progression. | Yes, women have been tracked through the Microsoft Excel model and logic was been found to be correct. |
| C4 | Unit testing: Have individual sub-modules of the computerized model been tested?  If yes, please provide information on the following aspects:  - Was a protocol that describes the tests, criteria, and acceptance norms defined beforehand?  - Please indicate where these tests and their outcomes are reported.  If no, please indicate why not.  Examples include but are not limited to: turning sub-modules of the program on and off; altering global parameters; testing messages (e.g., warning against illegal or illogical inputs), drop-down menus, named areas, switches, labelling, formulas and macros; removing redundant elements. | Drop down menus, Formulas and Macros have been tested. No, there was no prior protocol to describe these tests/criteria. Microsoft Excel sheet for the model indicates the use of these tests and its outcomes. |
|  | Part D: Operational validation (4 questions)  Part D discusses techniques used to validate the model outcomes. |  |
| D1 | Face validity testing (model outcomes): Have experts been asked to judge the appropriateness of the model outcomes?  If yes, please provide information on the following aspects:  - Who are these experts?  - What is your justification for considering them experts?  - To what extent did they conclude that the model outcomes are reasonable?  If no, please indicate why not.  Outcomes may include but are not limited to: (quality-adjusted) life years; deaths; hospitalizations; total costs. | Experts validated the outcomes for the model.   1. The Technical Advisory Committee for Health Technology Assessment in India validated the model outcomes. This committee comprises of clinicians, public health professionals, health economists’ and other senior experts with vast experience in their respective fields. 2. A senior health economist with more than five years of experience in health economic modelling having worked across various sectors in the Indian context validated the model outcomes.   The experts concluded that the model outcomes were appropriate.  The model outcomes and results were also presented to the Board for Health Technology assessment in India, under the Department of Health Research, Ministry of Health and Family Welfare, Government of India. The board comprises of senior professionals of the health sector nominated by the Indian government. The board has approved appropriateness of the outcome. |
| D2 | Cross validation testing (model outcomes): Have the model outcomes been compared to the outcomes of other models that address similar problems?  If yes, please provide information on the following aspects:  - Are these comparisons based on published outcomes only, or did you have access to the alternative model?  - Can the differences in outcomes between your model and other models be explained?  - Please indicate where this comparison is reported, including a discussion of the comparability with your model.  If no, please indicate why not.  Other models may include models that describe the same disease, the same intervention, and/or the same population. | Yes, comparison has been made with the only similar published outcome reported by a study from African setting, addressing the same problem. Though broad result in the compared study remains the same, there are multiple contextual and methodological dissimilarities between the studies, hence not comparable to a large extent. The comparision is reported in discussion section of the manuscript. |
| D3 | Validation against outcomes using alternative input data: Have the model outcomes been compared to the outcomes obtained when using alternative input data?  If yes, please indicate where these tests and their outcomes are reported.  If no, please indicate why not.  Alternative input data can be obtained by using different literature sources or datasets, but can also be constructed by splitting the original data set in two parts, and using one part to calculate the model outcomes and the other part to validate against. | No, alternative literature sources were not used to validate study findings as there was limited data available for the intervention under consideration. Available data was used to compute the model resutls. A detailed sensitivity analysis and value of information analysis however was undertaken to vary the input data to plausible extent and derive the value of reducing uncertaininty by generating further evidence. This validation is reported in discussion section of the manuscript. |
| D4 | Validation against empirical data: Have the model outcomes been compared to empirical data?  If yes, please provide information on the following aspects:  - Are these comparisons based on summary statistics, or patient-level datasets?  - Have you been able to explain any difference between the model outcomes and empirical data?  - Please indicate where this comparison is reported.  If no, please indicate why not. | No, not applicable. |
| D4A | Comparison against the data sources on which the model is based (dependent validation). | No, not applicable. |
| D4B | Comparison against a data source that was not used to build the model (independent validation). | No, not applicable. |
|  | Part E: Other validation techniques (1 question) |  |
| E1 | Other validation techniques: Have any other validation techniques been performed?  If yes, indicate where the application and outcomes are reported, or else provide a short summary here.  Examples of other validation techniques: structured “walk-throughs” (guiding others through the conceptual model or computerized program step-by-step); naïve benchmarking (“back-of-the-envelope” calculations); heterogeneity tests; double programming (two model developers program components independently and/or the model is programmed in two different software packages to determine if the same results are obtained). | No, other validation techniques were not performed. |
